# Brucellosis and Sacroiliitis Present as Acute Severe Hip Pain

**DOI:** 10.7759/cureus.61415

**Published:** 2024-05-31

**Authors:** Sarah M Alshamrani, Maryam S Alsharif, Fatimah M Khobrani

**Affiliations:** 1 Family Medicine, Ministry of National Guard Health Affairs, Yanbu, SAU; 2 Family Medicine, Ministry of Health, Yanbu, SAU; 3 Family Medicine, King Abdulaziz Medical City, Ministry of National Guard Affairs, Jeddah, SAU

**Keywords:** severe low back pain, malta fever, severe hip pain, sacroilitis, brucellosis

## Abstract

Brucellosis is a zoonotic disease that is endemic in Saudi Arabia. It is transmitted to humans through direct contact with infected animals or by consuming unpasteurized dairy products. A 36-year-old Saudi man presented with left hip pain, fever, and a history of consuming unpasteurized milk. Sacroiliitis was documented by positive results from serological tests (ELISA) and magnetic resonance imaging. Treatment consisted of non-steroidal anti-inflammatory drugs (NSAIDs), doxycycline, rifampicin, and IV gentamicin.

## Introduction

Brucellosis, or Malta fever, is a global infectious zoonotic disease caused by various Brucella species, which are nonmotile, facultative intracellular, aerobic, gram-negative coccobacilli [1].

In certain regions of Saudi Arabia, brucellosis is endemic and primarily spreads to people through contact with contaminated animals, consumption of animal products, or inhalation of contaminated aerosolized materials [2].

The disease has a wide range of clinical manifestations, and its symptoms can take months from exposure to infection to appear. The most frequent symptoms are fever, fatigue, headache, night sweats, weight loss, and, to a lesser extent, arthralgia and lymphadenopathy [3]. The infection can be mild or moderate and rarely progresses to chronic disease. Untreated infections can lead to severe complications and be fatal [4].

Sacroiliitis is a rare complication of brucellosis; it usually presents with severe low back pain and is often misdiagnosed as other inflammatory or mechanical disorders, leading to chronic joint problems and consequent marked disability [5].

## Case presentation

A 36-year-old man presented at the outpatient primary health care center of the National Guard Hospital in the Medina Region, Saudi Arabia, with complaints of left hip pain for a few days. His pain was aggravated by bending and was somewhat relieved by non-steroidal anti-inflammatory drugs (NSAIDs). There were no symptoms of fever, sweating, nighttime pain, morning stiffness, redness, or swelling. His pelvic X-ray was normal. However, his pain had worsened over the past three weeks. He arrived at the clinic in a wheelchair, unable to walk, with the pain radiating to the right hip joint and back. He then developed symptoms of fever and had a history of consuming unpasteurized milk.

A physical examination revealed localized tenderness of the joint and pain associated with ipsilateral straight leg raising. A neurological examination was normal. An infectious disease doctor was consulted, and it was recommended to send the patient to the emergency room for an MRI and a blood test to rule out septic sacroiliitis. The erythrocyte sedimentation rate (ESR) and C-reactive protein (CRP) levels were 33 mm/h and 45 mg/dL, respectively (Table 1). The hemogram and urinalysis were normal. Serological tests (ELISA for detecting anti-Brucella antibodies) were positive, but the blood culture was negative.

**Table 1:** Laboratory Test Results CRP: C-reactive protein; ESR: erythrocyte sedimentation rate.

Test	During 2 weeks	Reference Value
CRP	33	0.1~4.9 mg/L
ESR	45	0~15 mm/h

**Figure 1:**
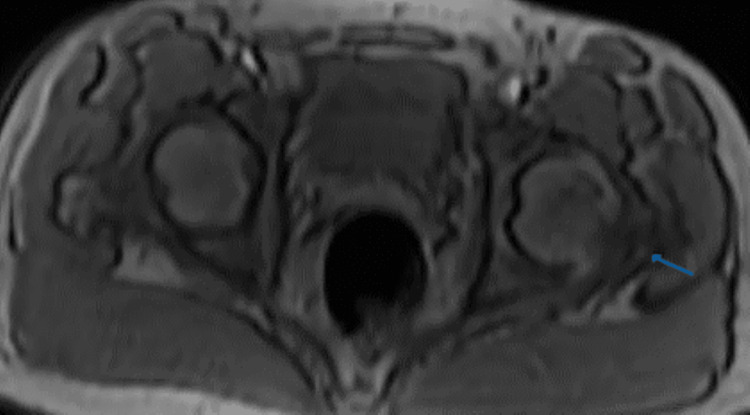
Both hip MRI findings: right sacroiliitis with trace fluid around the iliacus and multifidus muscles. Minimal left hip joint effusion.

An MRI of the sacroiliac joint revealed right sacroiliitis with trace fluid around the iliacus and multifidus muscles and minimal left hip joint effusion (Figure 1). The patient was admitted to the Infectious Diseases Department for 10 days of treatment with NSAIDs, doxycycline, rifampicin, and IV gentamicin. The patient was discharged home with oral rifampicin and doxycycline for eight weeks. Since discharge, the patient has been evaluated at regular intervals of two weeks for clinical and laboratory tests. The patient was successfully cured, with symptoms and complaints improving dramatically.

## Discussion

All around the world, brucellosis is an endemic zoonosis. Brucellosis is spread to humans through the consumption of unpasteurized milk-based dairy products or by touching contaminated animal products [6]. Between 20% and 85% of brucellosis patients have rheumatic problems, such as sacroiliitis and spondylitis, in addition to paraspinal abscesses, which result in excruciating back pain [7-10]. According to epidemiologic research, sacroiliitis is estimated to account for between 12% and 45% of musculoskeletal problems [10]. A previous study that reported three cases of brucella sacroiliitis found no distinctive symptomatology other than sacroiliitis, but it was characterized by an abrupt onset and very intense pain. This type of sacroiliitis causes relatively marked functional disability, especially early in the disease [6]. In the differential diagnosis of any patient presenting with sacroiliitis, brucellosis should be considered. Where the disease is endemic, there should be a high degree of suspicion [11].

Blood counts, ESRs, and CRP levels should all be part of routine laboratory testing. Blood counts in brucellosis cases are frequently characterized by mild anemia, thrombocytopenia, relative lymphocytosis, and leukopenia [12]. The isolation of Brucella species from blood or tissue samples establishes the definitive diagnosis of brucellosis. Bone marrow cultures are regarded as the gold standard for diagnosing conditions. However, blood is still the material most commonly used for bacterial culture, as obtaining bone marrow for culture is an invasive and painful procedure [13]. Studies have indicated that cultures negative for Brucella species do not rule out brucellosis because the proportion of cases with positive cultures varies from 15% to 70% [14, 15].

In any serological test for brucellosis, the presence of a rising antibody titer serves as additional evidence for the diagnosis of the disease. Based on the production of antibodies against lipopolysaccharides or other bacterial antigens, serologic methods for diagnosing brucellosis fall into two main categories [12]. A standard agglutination test yields significant titers above 1:160 and, in endemic areas, above 1:320 [12]. Radiographic abnormalities associated with musculoskeletal complications are known to arise later in the course of brucellosis. Therefore, it often happens that certain patients show no visible abnormalities on radiographs. The widening of the joint space and, later, the blurring of the subchondral bone are the most common radiographic findings of brucella sacroiliitis, and they usually manifest within two or three weeks [16, 17].

Technetium-99-methylene diphosphonate scintigraphy is a sensitive and practical diagnostic technique for identifying sacroiliac joint involvement. Additionally, a bone scan can detect other foci present in the body simultaneously. Sixteen cases of sacroiliitis have shown increased uptake in the sacroiliac joints. Recent perspectives suggest that MRI is superior to scintigraphy for evaluating active changes in the sacroiliac joint, citing sixteen cases due to its higher sensitivity in the early phases [18, 19].

In previous studies, all patients responded well to doxycycline and rifampicin therapy, though it is unclear if the condition is reactive or infectious. This can point to a sacroiliac joint-based focal infectious source, but it cannot rule out a reactive pattern, as other studies have indicated [20].

## Conclusions

Brucellosis is common in Saudi Arabia, but sacroiliitis is an uncommon presentation and can be missed and diagnosed as acute back pain or lumbar disc herniation. The diagnosis of brucella sacroiliitis should be considered in patients with acute back pain and fever.
